# Population Modelling in Affective Disorders

**DOI:** 10.1007/s40473-021-00229-6

**Published:** 2021-04-15

**Authors:** Erdem Pulcu

**Affiliations:** University of Oxford Department of Psychiatry, Warneford Hospital, Oxford, OX3 7JX UK

**Keywords:** Major depression, Computational modelling, Evolutionary biology, Temporal discounting, Social decision-making, Risk

## Abstract

**Purpose of Review:**

The prevalence of affective disorders is on the rise. This upward trajectory leads to a substantial personal and societal cost. There is growing body of literature demonstrating decision-making impairments associated with affective disorders, and more studies are using computational modelling methods to infer underlying mechanisms of these impairments from participant choice behaviour. However, lack of population modelling suggests that data resources may still be underutilised.

**Recent Findings:**

A number of recent studies associated major depression with abnormal risky decision-making as well as impairments in temporal discounting and social decision-making. These domains capture relevant aspects of real-life decision-making. Consequently, data from these studies can be used to define behavioural phenotypes for major depression.

**Summary:**

The manuscript describes a detailed proposal for population modelling to capture changes in the prevalence rate of major depression. The population modelling approach can also identify which decision-making domains can account for a larger part of impairments in psychosocial functioning and how behavioural interventions built on computational principles can target these to improve real-life psychosocial functioning in patient groups.

## Introduction

The last decade witnessed a so-called reproducibility crisis in *Psychology*, following a number of observed failures in replicating some of the experimental findings which were previously thought to be near factual [1–3]. Although social and cultural changes observed in the last century might be a contributing factor, a commonly acknowledged reason underlying the reproducibility crisis was over-estimating effect sizes from reasonably small cohorts [3]. Psychological sciences responded to the reproducibility crisis by requiring a higher number of replication experiments in order to validate effects before manuscripts can be recommended for publication, as well as promoting open data initiatives and source code sharing [4]. However, the consistency of findings in *Psychiatry* remained somewhat less scrutinised, mostly because identifying patients for studies (e.g. through rigorous structured clinical interviews) is laborious, and sample sizes are inherently small. The most likely reason for the lack of scrutiny is the assumption that a cognitive process of interest should be fundamentally different between patients and healthy volunteers, implying a large effect size. For example, a recent meta-analysis of reward processing studies in major depressive disorder suggested that achieving an effect size of *d* = 0.8 requires approximately 133 individuals per group, considerably much higher than the sample size of current psychiatric studies which average to 33 individuals per group [5]. Nevertheless, this changing landscape in how experimental work should be conducted in behavioural sciences influenced psychiatry to look beyond small and select laboratory-based cohort studies and consider associations between transdiagnostic traits (i.e. symptom domains which are not differentiating features of a single disorder but exist across different mental illnesses) in large scale population studies. One such approach is the Research Domain Criteria (RDoC) [6–8], aiming to pave the way for an integrative framework combining psychiatry and neuroscience for precision medicine. In this manuscript, I will argue that, despite a number of emerging findings demonstrating the usefulness of investigating associations between transdiagnostic psychiatric symptom categories and key behavioural outcome measures (e.g. risk-taking or ability to learn under uncertainty) in large-scale population studies [9, 10, 11•], data generated through this approach is underutilised. I propose that the field of psychiatry can benefit from computational methods of population modelling as a quantitative method of hypothesis testing. Here, by population modelling, I refer to the use of evolutionary biology models which use computer simulations to demonstrate how a population would change in time, based on relative success of the phenotypes (e.g. depression versus healthy) that exist in that population.

## A Need for a Paradigm Shift

Back in the 1980s and 1990s, population modelling, though limited by computational hardware, was a niche art that was, nevertheless, able to find explanations for the biological basis of some overtly puzzling and *costly* human behaviours such as interpersonal cooperation and altruistic punishment [12–18]. However, most probably due to a lack of necessary interdisciplinary will, [evolutionary] population models did not provide any empirical insight to questions such as “what is the biological basis of vulnerability to depression remaining in the genetic selection pool?” and such discussions remained theoretical [19–23]. In 2020, we have witnessed a surge in public interest in population modelling again, which proved to be instrumental in generating simulated outcomes that can inform public health policy in the face of the global SARS-CoV-2 pandemic [24]. For example, recently a Bayesian model of causality originating from neural sciences had been applied to estimate hidden parameters of a Gaussian distribution that could generate synthetic data mimicking daily coronavirus case reports as closely as possible [25]. Population modelling approaches allow making predictions about the future trajectory of an infectious disease. These models can also allow understanding of complex interactions between the parameters of the generative model such that it can be possible to identify treatment targets that can make the greatest impact at per unit cost of an intervention. However, although mental health conditions such as affective disorders are often associated with significant societal and personal costs (e.g. years lived with disability), and their prevalence is on the rise [26, 27], so far the relative urgency of the mental health crisis [28, 29] has not prompted the use of such outside-the-box thinking and interdisciplinary approaches like population modelling. For example, considering that a combination of hereditary (h^2^) and environmental influences increases lifetime risk factors for affective disorders [30], in a family of 2 adults and 2 children with one of the adults suffering from an affective disorder, the reproductive rate (conceptualised as *R*_0_ in infectious disease terms), of the psychiatric condition can be anywhere between 0 (in the case that none of the other family members experience any psychiatric problems) and 3 (in the case where the all of the family members eventually experience a psychiatric problem). Although this toy example illustrates the need for a paradigm shift for conceptualising the epidemiology of mental health disorders, it is also important to highlight that causes and prognosis of affective disorders are much more complex with considerable heterogeneity in symptom profiles.

## Screening for Future Treatments by Evolutionary Computational Models

One of the key problems that we are facing in the treatment of psychiatric conditions is identifying which treatments would work better and for which patient group. This problem is aggravated by a stagnation in drug discovery rates since 1960s and a reduction in interest from pharmaceutical companies until rapid antidepressant effects of NMDA receptor antagonists such as ketamine were identified in the past two decades [31, 32]. This therapeutic landscape gravely calls for novel behavioural interventions to step in. It is likely that novel behavioural interventions can be formulated from ground up, as behavioural interventions are less likely to have significant [cognitive] side effects [33], therefore can bypass some of the expensive and time-consuming phases of traditional drug discovery (e.g. phase I and II clinical trials). However, this can be possible insofar as we have a clear mechanistic understanding of affective symptoms [34] so that behavioural interventions targeting these mechanisms can be formulated or reverse engineered.

It is possible to conceptualise drug discovery in terms of an evolutionary process in which a number of candidate compounds compete for treatment efficacy and the winner takes all [35]. In the past decade, evolutionary approaches which are shown to speed up drug discovery [36••] were successfully implemented, for example, to identify antifungal compounds which can be repurposed to target carcinogenic tumours [37]. Below, I will argue that using an evolutionary computational framework can also speed up the discovery of novel behavioural interventions. I will give two examples: (i) a brief example conceptualising gambling addiction in terms of suboptimal reinforcement learning and value-based decision-making and (ii) a more detailed proposal for modelling behavioural phenotypes of major depressive disorder (MDD) in the population.

## Conceptualising Gambling Addiction as a Suboptimal Reinforcement Learning and Value-Based Decision-Making Phenotype

Excessive and persistent gambling is recognised as one of the manifestations of gambling disorder [38], a psychiatric condition that is more precisely characterised than affective disorders and known to be associated with behavioural traits such as impulsivity [39] and risk-taking [40–42]. One of the most lucrative games people can play in the UK is a national lottery which costs approximately £2.50 to play, with payouts usually between £14 and 180m depending on the number of rollouts, and 1 in ~14×10^7^ odds of winning. According to the classical economic theory, which posits that humans are concerned with reward maximisation and people should engage in reward-seeking behaviours only if they are expected to return a positive yield [43], no one should buy this lottery ticket until the rollover amount exceeds £350m. At this point, the expected outcome of the lottery is higher than the cost of play, and therefore every *rational actor* should play it. However, behavioural economic modelling has outlined nonlinear forms of subjective utility [44] and probability weighting [45–47] which corrupt these expected value computations. Suboptimal ways of inferring expected value may lead people to believe their choices are more desirable than they are, even when the expected value of a chosen gamble is objectively less than the associated cost. Although there is some preliminary evidence suggesting that a unique combination of risk-seeking and probability weighting preferences [expressed in terms of exponential utility and nonlinear exponential-logarithmic functions] may be associated with a competitive behavioural phenotype in the population [48], any suboptimal value computation is more likely to cause negative outcomes when individuals are concerned.

The other pathological aspect of the gambling disorder is the persistence of the behaviour. One aspect of this manifestation is known as the “gambler’s fallacy” which leads people to erroneously compute contingencies between independent events [49]. For example, a gambler continues to engage with new bets even when on a losing streak, thinking that loss events in the past indicate there should be more frequent win events in the future. This indicates that the person is simply not updating their beliefs about what outcome to expect from the next gamble (e.g. acknowledging how small the true odds of winning and the independence of draws). This is a textbook example of suboptimal reinforcement learning, such that the person is unable to update beliefs from negative prediction errors (i.e. the discrepancy between the expectation of winning money and the null outcome), pinpointing to very low learning rates. It is well-established that these cognitive processes can jointly be probed by binary decision-making tasks founded in reinforcement learning and value-based decision-making [47, 50, 51]. Despite striking cost effectiveness and quantitative precision of these behavioural computational measures [52] particularly relative to functional neuroimaging (fMRI) based approaches such as real-time neurofeedback [53], to the best of my knowledge, the former behavioural interventions have not yet been considered to have any clinical utility.

## Some Evolutionary Considerations for the Genetic Basis of Vulnerability to Depression

Before moving into a more detailed proposal about population modelling in major depression based on decision-making data, here I would like to touch upon some evolutionary biological considerations for the genetic basis of vulnerability to depression. Considering a widely accepted observation that depressive responses in wild mammals, for example, due to the loss of a cub, are usually transient due to natural selection pressures, it might only be possible to trace back the genetic basis of vulnerability to psychiatric conditions to a time when natural selection pressures on humans were substantially lower than the ancestral population [54]. Under these conditions, one can think of genetic basis of any vulnerability to psychiatric disorders in terms of mildly deleterious mutations which do not reduce fitness as steeply as strongly deleterious mutations that result in “genetic death” [55] and can have longer persistence times in the genetic pool [56]. This is in line with theoretical considerations, arguing that vulnerability to depression may have benefits in domains such as energy conservation [23]. In human populations of European ancestry, the ratio of male/female average mutation rate (i.e. for all degrees of deleterious mutations) is estimated to be 12:1 [57], raising the possibility that vulnerability to psychiatric conditions carried on by mildly deleterious mutations, that might be accumulating in the population, may be of paternal origin. It is noted that such accumulating mutations can lead to genetic deaths in future generations if favourable environmental conditions which relax natural selection cannot be sustained (e.g. due to climate change and other drastic environmental factors) [58]. Distribution of fitness effects (DFE) models [59] estimate that absolute fitness lost through accumulation of deleterious mutations can be as high as 0.02% per generation in individuals of European ancestry [60]. This estimate can be even higher if projections are made based on selection coefficients from invertebrates [54, 61]. However, as I will refer to in the next section, relative rather than absolute fitness is more relevant for understanding evolution of behavioural phenotypes by interpersonal competition and sexual selection.

## Describing Major Depression in Terms of a Multi-dimensional Decision-Making Phenotype for Population Modelling

**Fig. 1:**
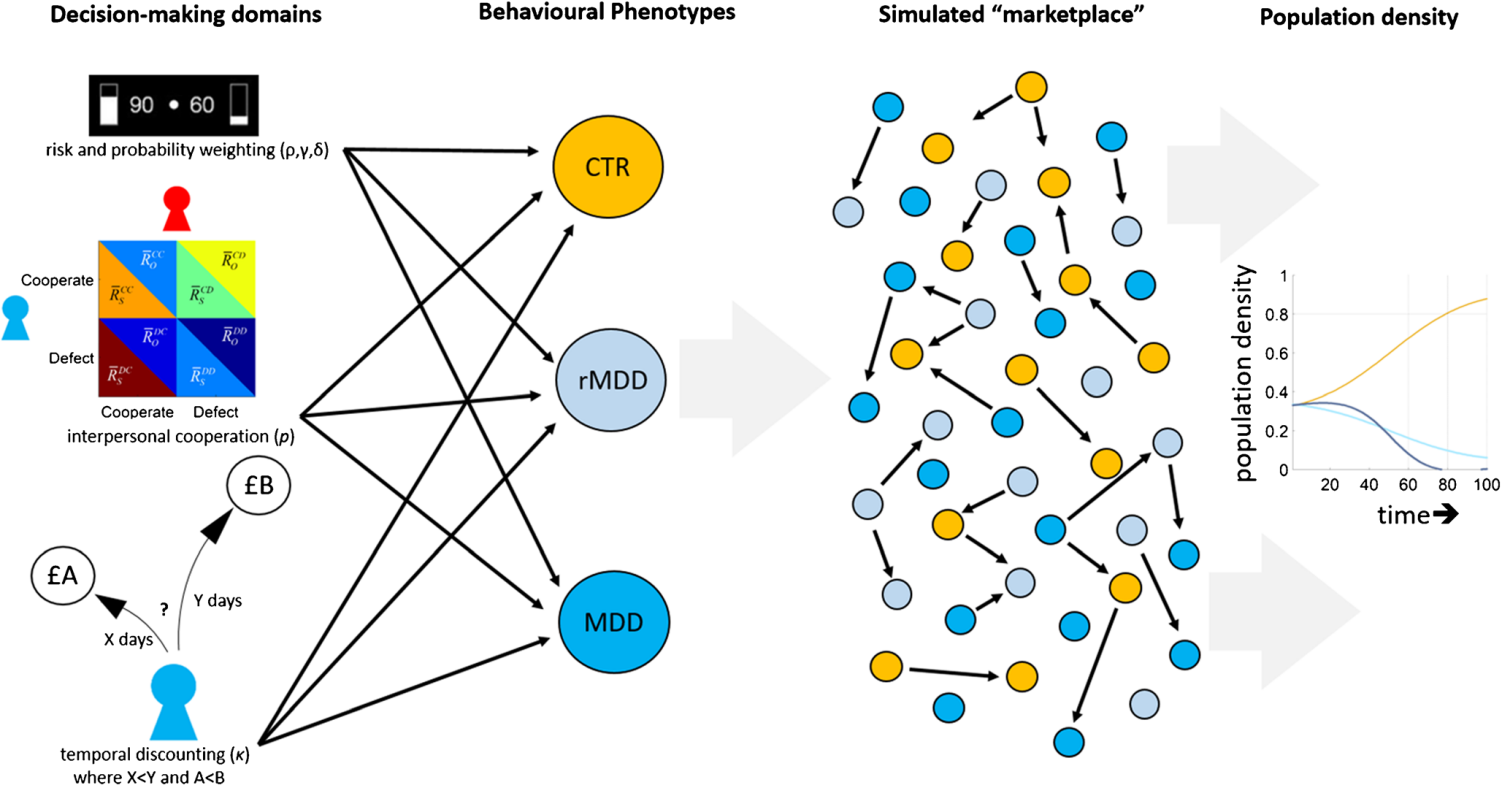
Key components of population modelling obtained by simulating behavioural interactions between agents. First, key decision domains relevant for a psychiatric condition need to be identified and parameterised (e.g. risky preferences commonly denoted by a power utility parameter ρ, probability weighting is expressed in terms of parameters γ, δ) in order to describe agent behaviour in terms of probabilistic strategies. In the case of major depressive disorder, these dimensions could be risky decision-making under uncertainty, interpersonal cooperation, and temporal discounting. These dimensions should be subjected to a weighted integration with respect to the sample sizes from which they have been drawn, in order to define behavioural phenotypes for each diagnostic group in an unbiased way, i.e. depression (MDD), vulnerability (rMDD), and healthy (CTR). Next, a simulated “marketplace” environment which consists of decision problems probing each of the domains which define the behavioural phenotypes needs to be constructed, such that agents compete and cooperate with each other to accumulate points, which are a measure of their evolutionary fitness. Finally, applying mathematically defined natural selection methods (e.g. linear Moran model [72]), transitions between the groups are allowed to observe how a certain behavioural phenotype can be optimal in the given environment

The field of decision neuroscience [62–64] and more broadly behavioural economics [65] relies on the assumption that decision-making scenarios involving management and distribution of tangible resources in laboratory and field experiments probe evolutionarily hardwired circuits. This link makes an a priori assumption that there would be a good degree of overlap between the human behaviour observed under experimental conditions (also including even more artificial environments such as inside an MRI scanner) and the decisions humans would make naturally in real life. As I have illustrated for gambling disorder, it is possible to apply the same understanding to describe major depressive disorder (MDD) in terms of a behavioural phenotype associated with various differences in decision-making in a number of key domains. Although many of these behavioural studies suffer from sample size limitations, as mentioned in the beginning of this manuscript, a number of previous studies suggested that MDD is associated with impairments in decision-making under uncertainty [66, 67], temporal discounting [68, 69], interpersonal cooperation and altruistic punishment [70, 71] (Fig. 1). Using existing parameters reported in the literature and weighing them proportionately by the sample size of each study (i.e. assigning a higher weight to the parameters reported from studies with larger sample sizes), it is straightforward to construct behavioural phenotypes for healthy as well as depression and remitted depression (rMDD) groups. Although this classification covers all individuals in a population with respect to MDD status, a potential limitation could be that this approach may capture a skewed representation of MDD solely based on patients willing to take part in research studies (i.e. a subgroup of altruistic patients who see utility in taking part in clinical research).

The next phase involves creating a simulated “marketplace” in which the agents, whose behaviour is governed by their probabilistic decision-making phenotypes, interact with each other and solve decision problems in return for rewards. In a simulated marketplace, the amount of rewards accumulated by each group would determine their competitive advantage over other phenotypes in the population, which in return will determine how the population evolves in time (Fig. 1). Here, the link between estimated evolutionary fitness and resulting population density (e.g. how much of the population remains vulnerable to MDD, i.e. the population density of the rMDD group) agrees with theories of sexual selection [73] and behavioural economic assumptions that I introduced earlier. This approach would also help objectively identifying conditions in which the behavioural phenotype associated with MDD would be the most competitive in the population and/or provide quantitative support for theoretical viewpoints associating vulnerability MDD with certain fitness benefits as I highlighted in the preceding section. In these models, individual agents can transition freely between being healthy, MDD and rMDD based on the relative fitness of their group compared with the population average. Over time, the proportion of the population that belongs to each group will reflect the overall fitness of that strategy, resulting in population densities of some of the groups gradually declining or reaching an equilibrium point. Here, a population equilibrium would demonstrate that decision phenotypes associated with MDD and vulnerability to MDD have enough competitive advantage over the healthy phenotype by which they remain in the selection pool. Increases in prevalence rates observed in real life may support this hypothesis that can be quantitatively tested by evolutionary simulation models. However, it is also possible to control such transitions between the groups by implementing an additional higher level coefficient that links mood and rewards received from the environment [74], such that a streak of negative outcomes can dampen the agent’s mood and increase vulnerability to depression. This proposal illustrates that the complexity of population modelling can be increased in the light of advances in our understanding of affective disorders, and different parameter combinations should be explored until a simulated population reflects the current prevalence rates of MDD in a population equilibrium.

In this methodology, simulating an interactive population that can accurately capture the current prevalence rates of MDD in the population would be an important endpoint. The simulated marketplace approach could also identify the aspects of depressed cognition which cause disadvantage to patients in real life, as well as identifying which aspects may give a competitive advantage. Then, it would also be possible to tweak the parameters of the MDD and the vulnerability phenotypes which underline decision-making impairments such that they can behave in a competitive manner. In descriptive terms, this would mean that although there might be differences in affective experience between the patients and healthy volunteers, the decision-making is no longer suboptimal, and consequently, real-life psychosocial and occupational functioning can remain uncompromised [75]. Theories of sexual selection posit that there is a positive correlation between competitive advantage and subjective/affective experience, suggesting a hypothesis that improvements in decision-making may also ameliorate depressive mood. Based on the scenario illustrated in Fig. 1, if the simulations pinpoint that abnormal temporal discounting (i.e. choosing between currently available and future rewards) accounts for the majority of lost fitness relative to the healthy phenotype, then training patients with MDD in optimal ways of solving temporal discounting problems should be the basis of cognitive bias modification interventions targeted to ameliorate psychosocial functioning impairments. This approach illustrates how it can be possible to formulate testable predictions based on simulated outcomes from a population model. I think this approach would be cost-efficient and quantitatively strong in terms of generating testable predictions.

## Concluding Remarks

In the current manuscript, I argued that it is possible to make greater use of existing behavioural data collected from populations with affective disorders, to construct informative population models which can help us to identify which cognitive processes may be the low hanging fruit in terms of the cost-to-benefit ratio of disseminating interventions. Doing so, I illustrated how gambling addiction and major depressive disorder can be conceptualised in terms of behavioural phenotypes that can capture the distinguishing features of these disorders in real life. I argue that the field of psychiatry as a whole should welcome more interdisciplinary collaborations in order to meet the public health challenges caused by rising prevalence rates of affective disorders.
